# Supplementary material for: Anti-Neuroinflammatory Effects of a Macrocyclic Peptide-Peptoid Hybrid in Lipopolysaccharide-Stimulated BV2 Microglial Cells
Source: Int J Mol Sci. 2024 Apr 18;25(8):4462. doi: 10.3390/ijms25084462 (PMC11049839; doi:10.3390/ijms25084462)
Supplement: Supplementary file 1 [file ijms-25-04462-s001.zip › ijms-2896944-supplementary.pdf]

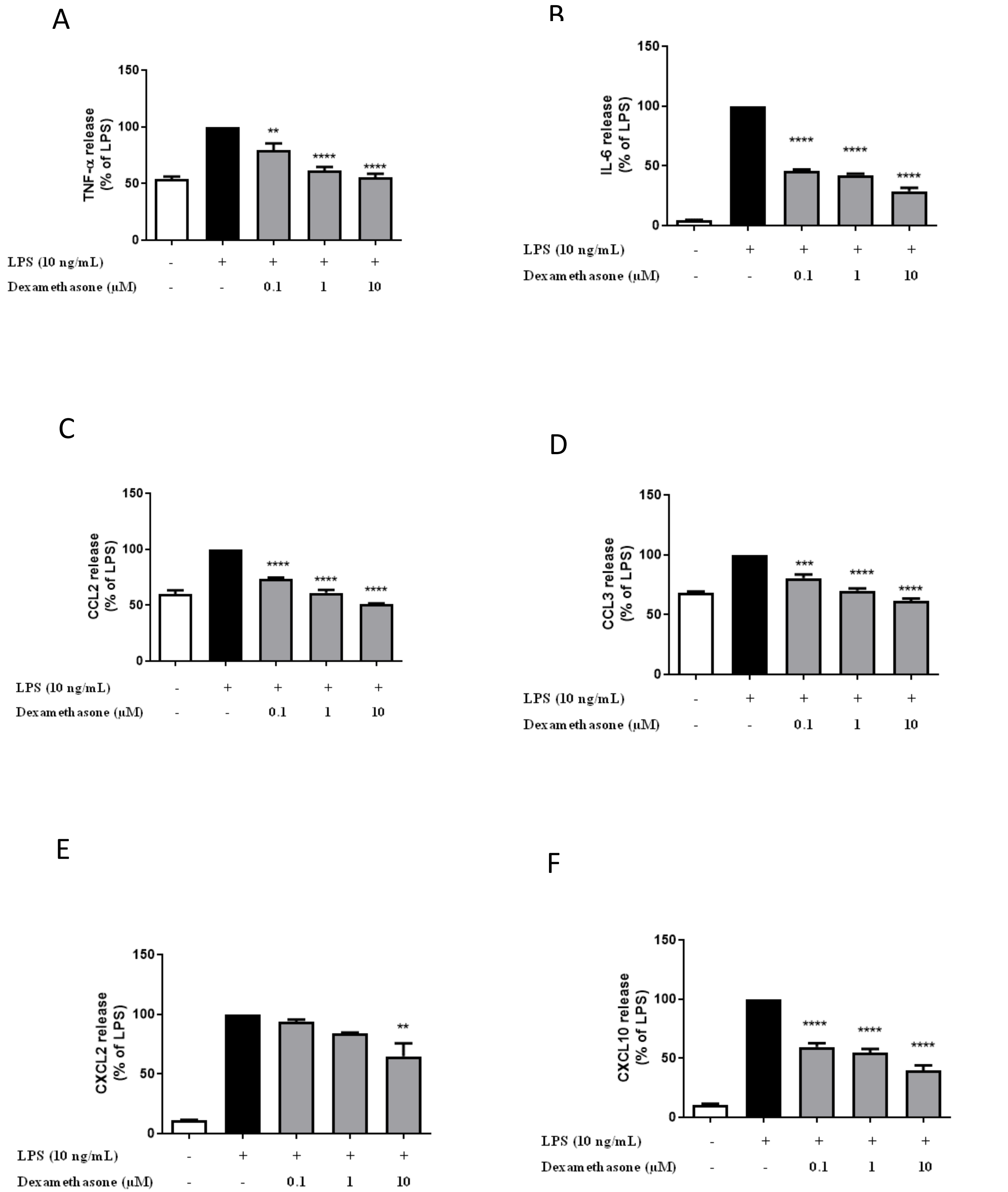

Supplementary Figure S1. Effects of dexamethasone on protein synthesis of TNF-α (A), IL-6 (B), CCL2 (C), CCL3 (D), CXCL2 (E), and CXCL10 (F) in LPS-stimulated BV2 cells. Cells were stimulated as described in the material and methods section. After 24 h of stimulation, supernatants were collected and the release of cytokines and chemokines was measured by ELISA. Values are presented as the mean ± SD of at least three independent experiments. Statistical analysis was performed using one-way ANOVA with Dunnett's post hoc tests and *$p<0.05$, **$p<0.01$, ***$p<0.001$, and ****$p<0.0001$.

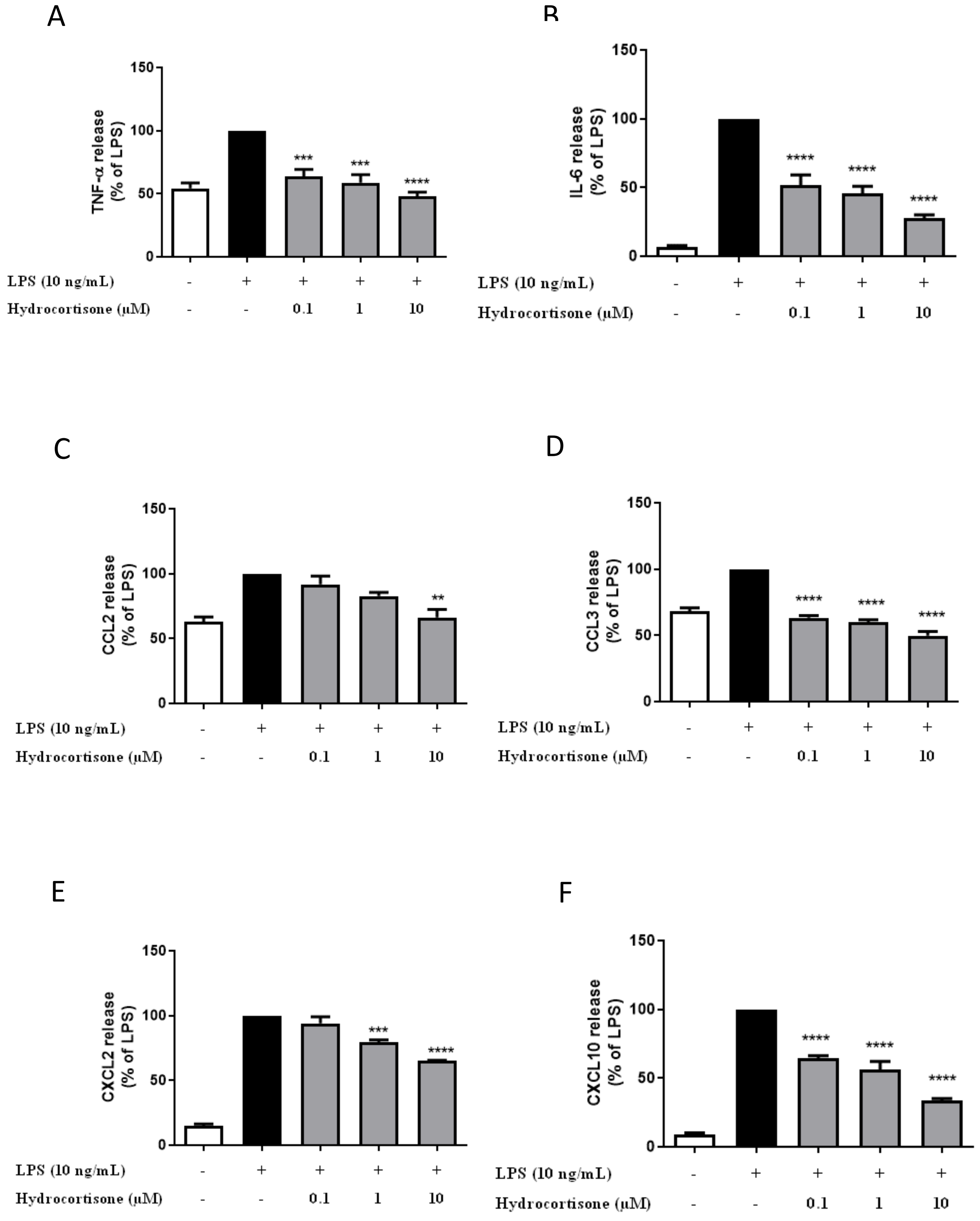

Supplementary Figure S2. Effects of hydrocortisone on protein synthesis of TNF-α (A), IL-6 (B), CCL2 (C), CCL3 (D), CXCL2 (E), and CXCL10 (F) in LPS-stimulated BV2 cells. Cells were stimulated as described in the material and methods section. After 24 h of stimulation, supernatants were collected and the release of cytokines and chemokines was measured by ELISA. Values are presented as the mean ± SD of at least three independent experiments. Statistical analysis was performed using one-way ANOVA with Dunnett's post hoc tests and $^{**}p<0.01$, $^{***}p<0.001$, and $^{****}p<0.0001$.
